# Vaccinia growth factor-dependent modulation of the mTORC1-CAD axis upon nutrient restriction

**DOI:** 10.1128/jvi.02110-24

**Published:** 2025-01-16

**Authors:** Lara Dsouza, Anil Pant, Blake Pope, Zhilong Yang

**Affiliations:** 1 Department of Veterinary Pathobiology, College of Veterinary Medicine & Biomedical Sciences, Texas A&M University 199063 , College Station, Texas, USA; The University of Arizona, Tucson, Arizona, USA

**Keywords:** vaccinia virus, poxvirus, mTORC1, CAD, pyrimidine, glutamine, asparagine, nutrition, metabolism, nutrient stress

## Abstract

The molecular mechanisms by which vaccinia virus (VACV), the prototypical member of the poxviridae family, reprograms host cell metabolism remain largely unexplored. Additionally, cells sense and respond to fluctuating nutrient availability, thereby modulating metabolic pathways to ensure cellular homeostasis. Understanding how VACV modulates metabolic pathways in response to nutrient signals is crucial for understanding viral replication mechanisms, with the potential for developing antiviral therapies. In this study, we establish the importance of *de novo* pyrimidine synthesis during VACV infection. We report the significance of vaccinia growth factor (VGF), a viral early protein and a homolog of cellular epidermal growth factor (EGF), in enabling VACV to phosphorylate the key enzyme CAD of the *de novo* pyrimidine pathway at serine 1859, a site known to positively regulate CAD activity. Although nutrient-poor conditions typically inhibit mTORC1 activation, VACV activates CAD via the mTORC1-S6K1 signaling axis in a VGF-dependent manner, especially upon glutamine and asparagine limitation. However, unlike its cellular homolog EGF, the VGF peptide alone, in the absence of VACV infection, has minimal ability to activate CAD. This suggests the involvement of other viral factors yet to be identified. Our research provides a foundation for understanding the regulation of a significant metabolic pathway, *de novo* pyrimidine synthesis during VACV infection, shedding new light on viral regulation under distinct nutritional environments. This study not only has the potential to contribute to the advancement of antiviral treatments but also improve the development of VACV as an oncolytic agent and vaccine vector.

**IMPORTANCE:**

Viruses often reprogram host cell metabolism to facilitate replication. How poxviruses, such as the prototype member, vaccinia virus (VACV), modulate host cell metabolism is not well understood. Understanding how VACV affects these metabolic pathways is key to learning about viral replication and developing antiviral treatments. This study highlights the importance of *de novo* pyrimidine synthesis during VACV infection. We found that the vaccinia growth factor (VGF), a viral protein similar to the cellular epidermal growth factor (EGF), helps VACV activate the enzyme CAD of the *de novo* pyrimidine pathway. Upon nutrient limitation, VGF is needed for the activation of CAD through mTORC1-S6K signaling. VGF peptide alone is unable to activate this pathway independent of infection, suggesting the involvement of other viral factor(s). Our research not only sheds light on how VACV regulates metabolism but also holds promise for improving VACV as a cancer treatment and vaccine.

## INTRODUCTION

Despite the eradication of smallpox, poxviruses remain a significant public health threat (1). This is exemplified by the current mpox (formerly known as monkeypox) outbreak, accentuating the ongoing challenges posed by poxviruses (2, 3). However, many poxviruses have also made significant contributions to fighting other diseases and advancing biotechnology, such as in vaccine development and oncolytic virotherapy (4). Employed as the vaccine for eradicating smallpox, Vaccinia virus (VACV) not only serves as the prototype member of the poxvirus family, but it is also the most extensively studied member of this family (5). Because of its close genomic resemblance to other orthopoxviruses with many encoded proteins that are almost identical among orthopoxviruses and as the vaccine for smallpox and mpox, VACV remains highly relevant for studying other highly pathogenic poxviruses (mpox, smallpox) (6). VACV has two copies of a gene encoding the early viral protein, vaccinia growth factor (VGF), which shares homology with cellular epidermal growth factor (EGF) and transforming growth factor-alpha (TGF-α) (7–9). Among the 118 early VACV genes, VGF is one of the most highly transcribed (10). VACV secretes VGF, binding to the EGF receptor (EGFR), thereby activating EGFR signaling and inducing proliferative effects in infected cells, consequently facilitating virus spread (11).

Viruses lack the intrinsic cellular machinery of metabolism and rely on host cells to provide metabolites and energy for synthesis and replication (12–14). An important yet understudied aspect of poxvirus-host interactions, which limits the potential to develop new prevention and therapeutic strategies, is the modulation of key pathways that regulate host metabolism upon poxvirus infection to produce the energy and metabolites necessary for viral replication (13, 15). We and others have previously reported that VACV actively alters host cell metabolism (16). We demonstrated elegant mechanisms that identified the requirement of VGF to increase TCA cycle intermediates via STAT3 and reprogramming of fatty acid metabolism upon VACV infection (17, 18).

Nucleotides, in addition to their fundamental role in DNA and RNA assembly, also contribute to protein glycosylation, phospholipid synthesis, lipogenesis, and the regulation of innate immune responses (19–21). Cells can produce nucleotides via either the *de novo* synthesis pathway or salvage pathways (22). In non-proliferative resting cells, which do not require high amounts of metabolites, nucleotides are sourced from salvage pathways. These pathways essentially involve the recycling of nucleotides obtained from DNA and RNA degradation or the uptake of nucleosides or nucleobases from the extracellular space (23, 24). During periods of heightened nucleotide demand, the *de novo* synthesis pathway is activated, facilitating nucleotide synthesis from amino acids and glucose, thereby supporting efficient viral replication (23, 24). Recent studies suggest that some viruses enhance the synthesis of nucleotides to facilitate replication (25–27). Earlier research has demonstrated that VACV replication was reduced when treated with N-phosphonacetyl-L-aspartate (PALA), an inhibitor of the *de novo* pyrimidine pathway (28). CAD (carbamoyl-phosphate synthetase II, aspartate transcarbamoylase, dihydroorotase), the first key enzyme of the *de novo* pyrimidine pathway, is known to be regulated by upstream growth factor MAPK signaling (22, 29–31). However, how VACV regulates pyrimidine metabolism remains unknown.

Given the need for an increased supply of nucleotides to facilitate efficient VACV replication, and with increasing evidence emphasizing the significance of growth factor signaling in activating pyrimidine synthesis, we hypothesized that VGF, VACV’s homolog to EGF, plays an important role in the regulation of pyrimidine synthesis upon VACV infection. Additionally, we had earlier demonstrated that VACV prefers glutamine over glucose to achieve efficient replication (15). This preference stems from the fact that asparagine, crucial for viral protein synthesis, is synthesized from glutamine, in addition to exogenous supply (32). Glutamine plays a critical role in the functionality of rapidly proliferating cells, as numerous cellular processes rely on glutamine (33). When exogenous glutamine is unavailable, cancer cells are capable of synthesizing glutamine *de novo*, with the exception of asparagine (33–36). Our group had previously elucidated the critical role of asparagine in facilitating VACV’s effective replication upon glutamine limitation, highlighting a specific metabolic vulnerability that can be leveraged for targeted antiviral therapeutic interventions (32). We have now further expanded upon this study by elucidating how VACV adapts to glutamine and asparagine restriction by utilizing its early protein VGF to phosphorylate CAD of the pyrimidine synthesis pathway. Additionally, we report that VACV modulates mTORC1 to post-translationally modify CAD through VGF during nutrient insufficiency. Emerged as a central regulator of metabolism, mTORC1 incorporates growth factor signals and nutrient availability to regulate both anabolic and catabolic processes (37, 38). Understanding the diverse downstream impacts of mTORC1, particularly in the context of VACV infection, remains limited, despite its potential to substantially affect metabolic and growth-related processes.

In this study, we report that although amino acid limitation in uninfected cells fails to activate growth factor signaling, VACV, even in conditions of nutrient restriction, can activate the mTORC1 pathway through its viral growth factor VGF. Despite nutrient insufficiency, this emphasizes the virus’s ability to leverage growth factor signaling. Our results offer insights into how diverse nutritional environments serve as cues for VACV to utilize/regulate signaling pathways differently, facilitating its efficient replication. This study advances our understanding of viral adaptation to nutrient stress, offering knowledge that could inform the development of combination therapies targeting both metabolic vulnerabilities and associated signaling pathways, thereby improving therapeutic efficacy. Cancer cells are known to survive in nutrient-limited environments, and VACV has been developed as a promising oncolytic agent (34, 39). Our research investigating how VACV modulates cellular signaling in response to nutrient deprivation has the potential to not only inform the design of combinatorial therapeutic strategies targeting poxviruses but also further enhance VACV’s efficacy as an oncolytic tool (39, 40).

## MATERIALS AND METHODS

### Cell culture

Human foreskin fibroblasts (HFFs) were maintained in Dulbecco’s minimal essential medium (DMEM; Fisher Scientific), and BS-C-1 cells (ATCC CCL-26) were maintained in Eagle’s minimal essential medium (EMEM; Fisher Scientific). Both media were supplemented with 10% fetal bovine serum (FBS; Peak Serum), 2  mM glutamine (VWR), 100  U/mL of penicillin, and 100  µg/mL streptomycin (VWR).

Specialized DMEM (1× DMEM, no glucose, no glutamine, no phenol red; Fisher Scientific; Cat #A1443001) was used in indicated experiments.

For protocols performed in the absence of serum starvation, HFFs were infected with wildtype (WT-VACV) or mutant VACV with VGF gene deleted (vΔVGF) upon reaching confluence. The infections were carried out using 1× DMEM supplemented with 2% dialyzed FBS (Gibco) and either 2 mM glutamine (Quality Biological) or 2 mM asparagine (Sigma-Aldrich) as specified for 8 h at the indicated MOI.

For protocols using serum-starved conditions, HFFs were initially starved using 1× DMEM supplemented with 0.1% dialyzed FBS and either 2 mM glutamine or 2 mM asparagine as specified for a period of 48 h followed by infection with WT-VACV or vΔVGF for 8 h with fresh media containing the same supplements as those used during starvation.

For protocols performed using glutamine and asparagine restriction conditions, HFFs were initially starved for approximately 36 h in 1× DMEM supplemented with 0.1% dialyzed FBS and 2 mM asparagine. Sixteen hours before infection, asparagine supply was restricted, by replacing media with 1× DMEM supplemented with 0.1% dialyzed FBS and glucose only. The total starvation period for the HFF cells was 48 h, followed by infection for 8 h with fresh media containing the same supplements as those used for the 16 h asparagine-restricted starvation. All cells were cultured in a humidified incubator set at 37°C with 5% CO_2_.

### Viruses and infection

The VACV Western Reserve (WR) strain (ATCC VR-1354) was used for the generation of WT-VACV and vΔVGF virus as described previously (18). The stage-specific recombinant VACVs, which express *Gaussia* luciferase under VACV early, intermediate, or late promoters (vEGluc, vIGluc, and vLGluc, respectively) were used in this study. Amplification, purification, and titration of these viruses were conducted following established protocols described elsewhere (41). Virus infection was carried out using the indicated multiplicity of infection (MOI) in regular DMEM supplemented with 2.5% FBS, 2 mM glutamine, and 1 g/L glucose (unless specified otherwise) followed by plaque assay.

### Plaque assay

BS-C-1 cells were infected with WT-VACV, and the medium was replaced with EMEM supplemented with 0.5% methylcellulose (Fisher Scientific) 1 h post-infection (hpi). After 48 h, the medium was discarded and replaced with 0.1% (wt/vol) crystal violet solution dissolved in 20% ethanol for 10 min. Plaques were counted, and titers were determined using a previously described method (42).

### Cell viability assay

The cell viability assay was conducted using the trypan blue exclusion method, following previously established procedures (43). HFFs were cultured in a 12-well plate, followed by trypsinization with 200 µL of trypsin and consequently suspended in 500 µL of DMEM. The cells were centrifuged at 1,000 × *g* for 5 min at 4°C. The supernatant was discarded, and the pellet was resuspended in 30 µL of DMEM. Additionally, 10 µL of the suspended pellet was mixed with 10 µL of 0.4% trypan blue (VWR), and 10 µL of this mixture was added to the dual chamber counting slides, and cell viability was measured using an automatic cell counter (Bio-Rad).

### *Gaussia* luciferase activity assay

A luminometer and the Pierce *Gaussia* luciferase flash assay kit (Thermo Scientific) were used to measure *Gaussia* luciferase activity as per the manufacturer’s instructions.

### Quantitative PCR

DNA was extracted utilizing the EZNA Blood DNA Kit. Relative levels of viral DNA were quantified through the CFX96 real-time PCR instrument (Bio-Rad, Hercules, CA) and the All-in-one 2× qPCR mix (GeneCopoeia), utilizing specific VACV primers targeting the C11R gene. As internal references, primers for the 18S rRNA gene were used. The initial denaturation step was started at 95.0°C for 10 min followed by 40 cycles of denaturation at 95°C for 10 s, annealing at 53°C for 30 s, and extension at 72°C for 15 s. The sequences for the primers used are as follows:

C11p FW: AAACACACACTGAGAAACAGCATAAA

C11p Rev: ACTATCGGCGAATGATCTGATTATC

18S rRNA FW: CGA TGC TCT TAG CTG AGT GT

18S rRNA Rev: GGT CCA AGA ATT TCA CCT CT

### Metabolic profiling

Metabolic profiling was performed according to established protocols as described previously (32). Four biological replicates (T-175 flask per replicate) for each condition were used. On reaching 95% confluence, HFF cells were briefly rinsed twice with 1× PBS at 37°C and subsequently infected with either WT- VACV or vΔVGF at a multiplicity of infection (MOI) of three in the designated media for 8 h.

At 8 hpi, cells were collected by scraping, followed by two washes with ice-cold 1× PBS. The cell pellets were then resuspended in methanol extraction solvent and stored at −80°C until shipment to Metabolon. Samples were prepared using the MicroLab STAR system for QC, methanol precipitation, and TurboVap for solvent removal. Analysis was conducted using Waters ACQUITY UPLC and a Thermo Q-Exactive mass spectrometer (HESI-II source, Orbitrap analyzer at 35,000 resolution). Data were normalized to Bradford protein concentration, log-transformed, and missing values imputed. A one-way ANOVA assessed differences between samples.

### Inhibitors and antibodies

The chemical inhibitors leflunomide (Cat #1247) and rapamycin (Cat #s1039) were purchased from Selleck Chemicals. Pyrazofurin (Cat #SML1502) was purchased from Sigma Aldrich. PALA was acquired from the Drug Synthesis and Chemistry Branch, Development Therapeutics Program, Division of Cancer Treatment and Diagnosis, National Cancer Institute. Antibodies targeting CAD at serine1859 (Cat #12662), total CAD (Cat #11933), DHODH (Cat #80981), mTOR (Cat #2972S), p-mTOR ser2448 (Cat #2971), S6K1 T389 (Cat #9234), and total S6K1 (Cat #2708) were purchased from Cell Signaling Technology. CAD threonine 456/pCPS2 (Cat #sc377559), anti-glyceraldehyde-3-phosphate dehydrogenase (anti-GAPDH) (Cat #sc-365062), anti-alpha-actin (Cat# sc32251, and anti-beta-tubulin (Cat#, sc-55529 antibodies were sourced from Santa Cruz Biotechnology.

### Western blotting analysis

Western blotting analysis was conducted according to the procedure described elsewhere (18). Briefly, samples were prepared using NP-40 lysis buffer, followed by the addition of 100 mM dithiothreitol (DTT) as a reducing agent and sodium dodecyl sulfate as the loading buffer. Following boiling at 95°C for 5 min, the samples were loaded onto SDS–PAGE gels. The gels were subsequently transferred for 10 min onto a polyvinylidene difluoride membrane (PVDF) using the Trans-Blot turbo transfer system (Bio-rad). The membranes were blocked using blocking buffer prepared using 5% milk (Alkali Scientific) in 1× TBST for 1 h at room temperature, followed by overnight incubation with the primary antibody in blocking buffer (5% milk in 1× TBST) at 4°C or 1 h at room temperature. The membrane was washed three times for 10 min each with 1× TBST and incubated with a horseradish peroxidase-conjugated secondary antibody (Cell Signaling Technology; Cat #7074S) for 1 h at room temperature. Membranes were developed using Thermo Scientific SuperSignal West Femto Maximum Sensitivity Substrate (Thermo Fisher; Cat #34095) and imaged using Chemiluminescent imager (Azure 300). Antibodies were stripped from the membrane using Restore Plus western blotting stripping buffer (Fisher Scientific, Cat #46430) for Western blotting analysis with another primary antibody.

We used GAPDH as a loading control in many of the Western blotting analyses. However, CAD, being a large protein, at times required a lower-percentage gel (6%) for best detection. GAPDH detection by Western blotting in low-percentage gel is challenging as it is relatively small (~35 kDa). We used alpha-actin or beta-tubulin (with relatively large molecular weights) as alternative loading controls in some instances.

### VGF peptide expression, purification, and treatment

The sequence for the cleaved, secreted portion of the VGF peptide was obtained from NCBI YP_232891.17–9 A signal peptide (from the Mouse IgG antibody) was added at the N terminal region (red, cleaved after secretion), and a 6×His tag at the C terminal region (MGWSCIILFLVATATGVHSDSGNAIETTSPEITNATTDIPAIRLCGPEGDGYCLHGDCIHARDIDGMYCRCSHGYTGIRCQHVVLVDYQRSENPNTHHHHHH) was expressed from plasmid encoding *de novo* synthesized gene and purified using a mammalian expression system using the HD CHO-S cell line at GenScript (44, 45). This process involved cloning the peptide’s secreted sequence with 6×His tag encoded gene into the vector pcDNA3.4, followed by plasmid preparation, TurboCHO 2.0 expression, and one-step purification using Ni-NTA. The purity of the peptide was assessed by SDS-PAGE Coomassie Blue staining and SEC-HPLC. A ACQUITY UPLC H-CLASS system for high-resolution separations was used in the SEC-HPLC. The samples were diluted in buffer suitable for the mobile phase (0.1 mol/L Na₂SO₄ in 0.118 mol/L PBS (pH 6.7 ± 0.3), and an AdvanceBio SEC 200A column was used for size-based separation. Prior to injection, the sample was desalted, and the sample volume of 14.5 µL was used. The peptide was dissolved in 1× PBS (pH 7.2) for storage.

### Statistical analysis

The data presented represent the mean of at least three biological replicates unless mentioned otherwise. Error bars depict the standard deviation of the experimental replicates.

A Student’s *t*-test was conducted to assess any significant differences between the two means, and a one-way ANOVA was used to compare significant differences between multiple groups. We utilized the following symbols to denote statistical significance: ns, *P*  >  0.05; *, *P*  ≤  0.05; **, P ≤ 0.01; ***, P ≤ 0.001; ****, P ≤ 0.0001. Figures were prepared using GraphPad version 10.2.3, and schematics were created using BioRender.com.

## RESULTS

### *De novo* pyrimidine synthesis is required for efficient VACV replication

**Fig 1:**
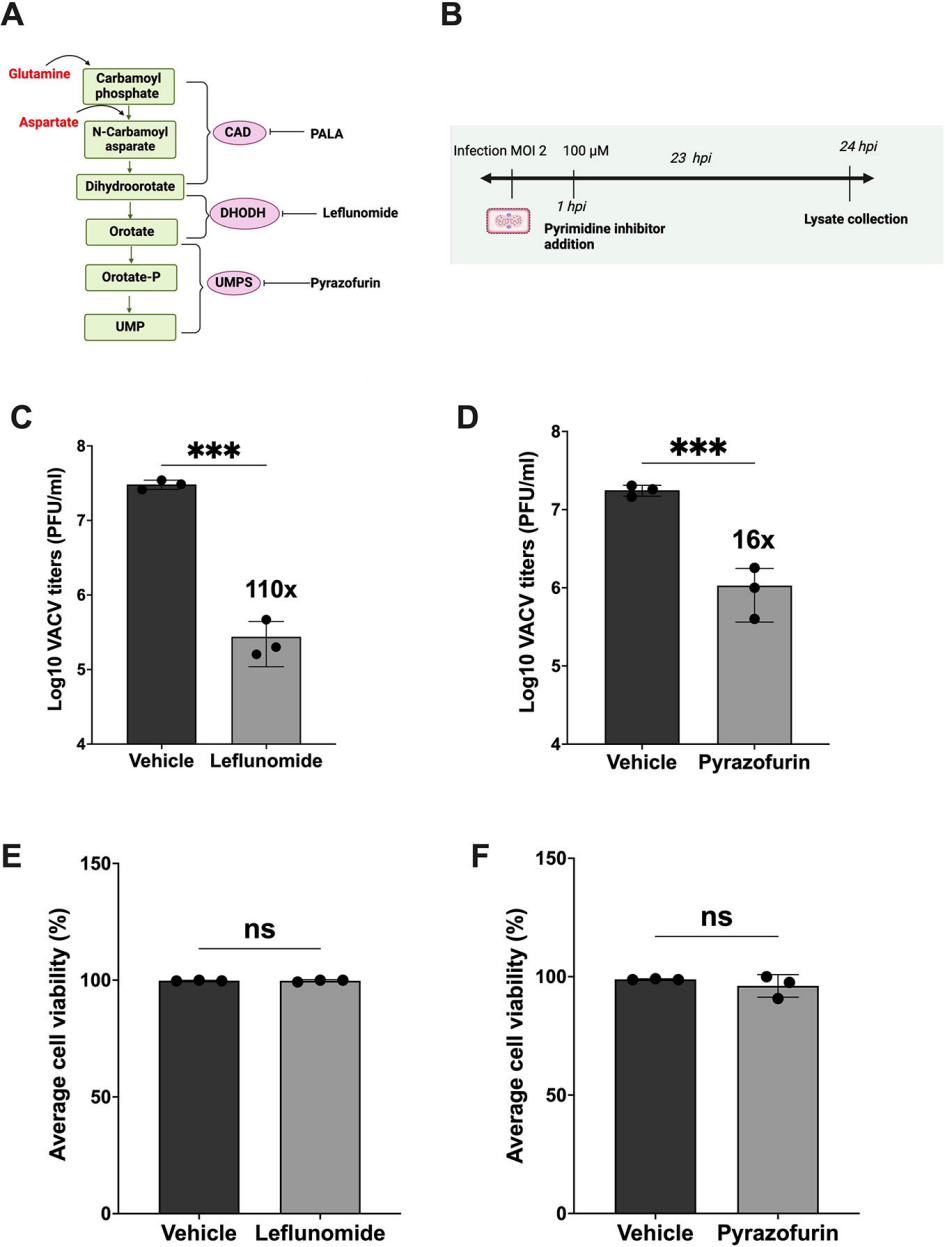
Inhibition of *de novo* pyrimidine pathway significantly reduces VACV replication. (**A**) An overview of the *de novo* pyrimidine pathway with compounds inhibiting key enzymes of this pathway. (CAD, carbamoyl phosphate synthetase II, aspartate transcarbamoylase, dihydroorotase) (DHODH, dihydroorotate dehydrogenase) (UMPS, uridine monophosphate synthetase) (PALA , N-phoshponacetyl-L-aspartate). (**B**) Timeline of infection, the addition of inhibitors, and collection of lysate (C and D) HFFs were infected with VACV at an MOI of 2 and treated with the indicated compounds 1 hpi at a concentration of 100 µM. Cell lysates were collected at 24 hpi for plaque assay using BSC-1 cells. (E and F) HFFs were treated with leflunomide or pyrazofurin (100 µM) and cell viability was measured using trypan blue staining after 48 h treatment. Error bars represent the standard deviation of at least three biological replicates. ns, *P* > 0.05; ***, P ≤ 0.001. Statistical analysis for the difference between the two means was performed using the student’s *t* test.

The *de novo* synthesis of pyrimidines is initiated with glutamine and the trifunctional enzyme CAD, leading to the production of dihydroorotate, which is subsequently oxidized to orotate by dihydroorotate dehydrogenase (DHODH). Orotate is subsequently phosphorylated by the bifunctional enzyme UMP synthase (UMPS) to yield uridine monophosphate (UMP). UMP serves as the primary metabolite for pyrimidine synthesis (30, 46) (Fig. 1A).

Previous studies have demonstrated that PALA, a selective inhibitor targeting the CAD enzyme, significantly attenuates VACV replication *in vitro* (28). Expanding upon these findings, we sought to elucidate the role of *de novo* pyrimidine synthesis in VACV infection by employing selective inhibitors against the remaining enzymes of this pathway, DHODH and UMPS, and assessing their impact on VACV replication in HFFs (47–50). In cells infected with VACV at an MOI of 2, we administered leflunomide (a DHODH-specific inhibitor) or pyrazofurin (a UMPS-specific inhibitor) for 1 hpi, and the cell lysates were collected at 24 hpi. Our observations revealed a pronounced and statistically significant reduction in VACV titers in the treated samples compared with the vehicle controls, with titers decreasing by 110-fold in leflunomide-treated cells and 16-fold in pyrazofurin-treated cells. Importantly, these inhibitory concentrations did not negatively impact cellular viability (Fig. 1B through F). These results confirm the importance of *de novo* pyrimidine synthesis during VACV replication.

### *De novo* pyrimidine pathway is required for VACV DNA replication and subsequent intermediate and late gene expression

**Fig 2:**
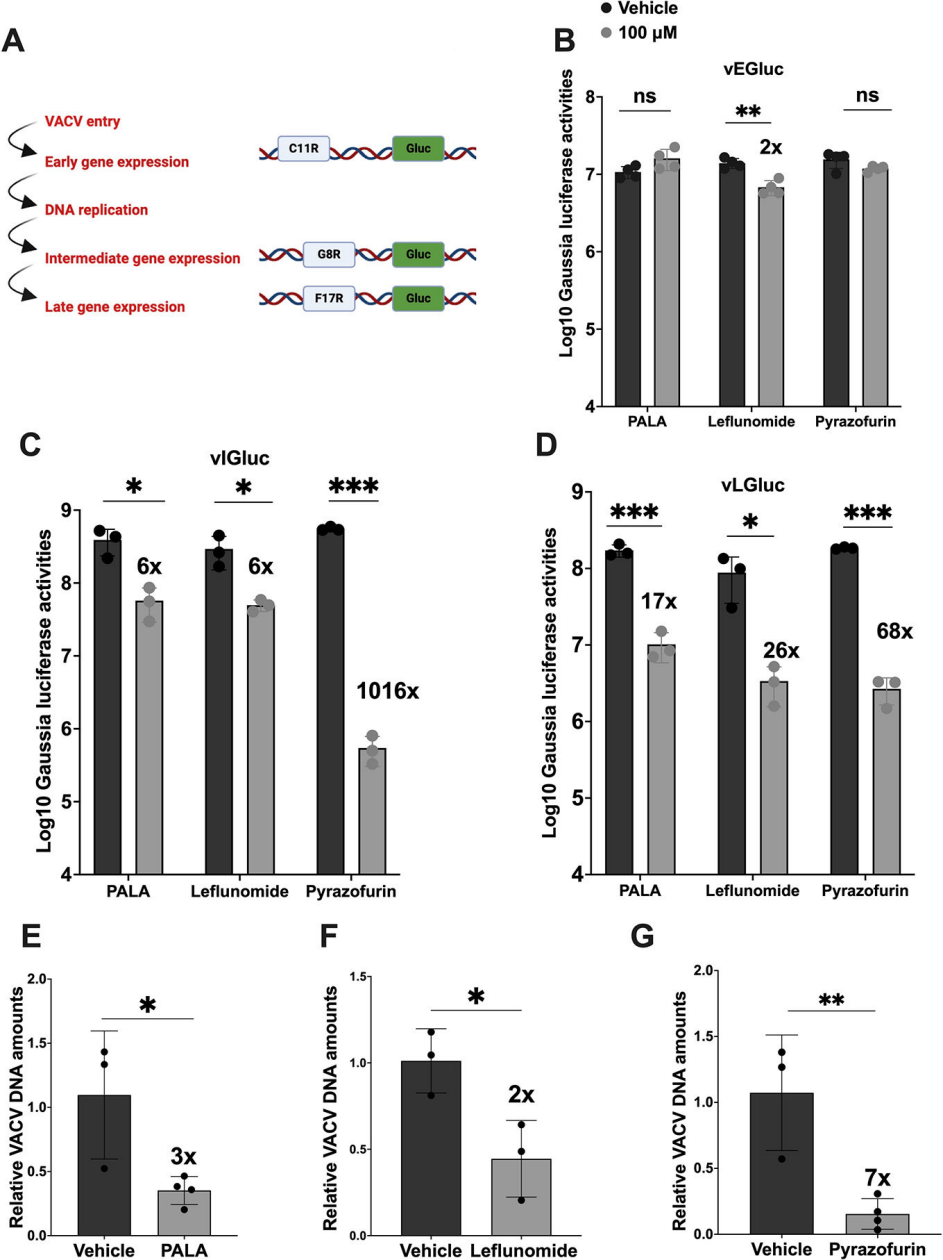
*De novo* pyrimidine pathway is required for VACV DNA replication and subsequent intermediate and late gene expression. (**A**) Schematic of the temporal expression pattern of VACV genes with recombinant VACV containing stage-specific *Gaussia* reporter genes. (B, C, and D) Inhibition of *de novo* pyrimidine pathway reduces VACV intermediate and late gene expression. VACV gene expression was quantified by measuring *Gaussia* luciferase activities using reporter VACVs expressing *Gaussia* luciferase under early (vEGluc, **B**), intermediate (vIGluc, **C**), and late (vLGluc, **D**) promoters, respectively. HFFs were pre-treated with the indicated compounds for approximately 16 h prior to infection and subsequently infected with the indicated VACV at an MOI of 2, followed by treatment with the indicated compounds at 1 hpi. *Gaussia* luciferase activities were measured at 4 hpi for early gene expression and 8 hpi for intermediate and late gene expression. (E, F, and G) Inhibition of *de novo* pyrimidine pathway reduces VACV DNA levels. HFFs were pretreated with indicated pyrimidine synthesis inhibitors 16 h prior to infection and subsequently infected with WT-VACV virus at an MOI of 2, followed by treatment with indicated *de novo* pyrimidine pathway inhibitors at 1 hpi. Cell lysates were collected at 8 hpi. DNA was extracted and relative viral DNA levels were determined using real-time PCR with VACV-specific primers. Error bars represent the standard deviation of at least three biological replicates. ns, *P* > 0.05; *, P ≤ 0.05; **, P ≤ 0.01; ***, P ≤ 0.001. Statistical analysis between two means was performed using the student’s *t* test.

VACV gene expression is temporally regulated with early gene expression initiated upon viral entry into the host cell. Subsequently, the expression of intermediate and late genes is contingent upon the completion of viral DNA replication. This latter stage is regulated by transcription factors that are products of early and intermediate gene expressions, respectively (51). To further test which stage of VACV replication is influenced by the *de novo* pyrimidine pathway, we employed three recombinant VACVs, each harboring a Gaussia luciferase reporter gene under the control of distinct viral promoters, indicative of early (C11R), intermediate (G8R), or late (F17R) expression stages (Fig. 2A). Luciferase activities were quantitatively measured at 8 hpi for the assessment of intermediate and late promoter-driven gene expression, and at 4 hpi for early gene expression. Despite observing a marginal decrease in luciferase activities in leflunomide-treated samples relative to vehicle controls, the difference in early gene expression between vehicle-treated and inhibitor-treated VACV-infected cells was not pronounced (Fig. 2B). Conversely, a notable 6-fold decrease in intermediate gene expression was recorded in infected cells upon treatment with inhibitors targeting the CAD or DHODH enzymes (Fig. 2C).

A more drastic reduction, approximately 1,000-fold, in intermediate gene expression was observed with UMPS inhibition (Fig. 2C). In addition, significant decreases in late gene expression were recorded upon pathway inhibition with each of the three inhibitors individually, with fold reductions of approximately 17 (PALA), 26 (leflunomide), and 68 (pyrazofurin), respectively (Fig. 2D).

Given that VACV’s intermediate and late gene expressions are dependent upon viral DNA synthesis, the observed reductions in gene expression can be partially attributed to diminished viral DNA levels and/or impeded viral RNA synthesis. Further analysis on the effect of *de novo* pyrimidine pathway inhibition on VACV DNA quantities revealed a decrease in viral DNA levels, with a ~ 3-fold reduction upon PALA treatment, ~2-fold decrease with DHODH inhibition, and ~7-fold reduction upon UMPS inhibition in HFF cells (Fig. 2E through G). Collectively, these results affirm the important role of *de novo* pyrimidine synthesis in VACV replication, commencing from the DNA replication stage.

### VGF-dependent increase in the *de novo* pyrimidine pathway intermediates upon VACV infection

To assess whether VACV modulates *de novo* pyrimidine synthesis, we conducted LC-MS profiling, comparing the *de novo* pathway intermediates between uninfected and WT-VACV-infected HFFs. Furthermore, our objective was to identify the specific viral factor responsible for the regulation of pyrimidine synthesis upon infection. Given the temporal gene expression pattern of VACV and that VACV DNA replication commences around 2 hpi, a process that is temporally aligned with the early stage of gene expression, suggests that an early viral factor facilitates pyrimidine synthesis.

With evidence highlighting the significance of EGF in modulating pyrimidine metabolism, we hypothesized that VGF, a cellular homolog of EGF and an early vaccinia viral factor, plays a critical role in the regulation of pyrimidine synthesis (29–31). To test our hypothesis, we employed a recombinant VACV with VGF gene deleted (vΔVGF), aiming to investigate the role of VGF absence on the levels of *de novo* pyrimidine pathway intermediates upon VACV infection (16). Additionally, building on our previous work to identify how glutamine and asparagine can influence the regulation of metabolic pathways by VACV, we conducted the metabolic profiling under two distinct media conditions: medium containing asparagine or glutamine. Utilizing an asparagine-containing medium that is independent of glutamine also allowed for an investigation into the metabolic impacts exerted by VACV without the confounding effects introduced by glutamine availability, a central carbon source for metabolic pathways.

**Fig 3:**
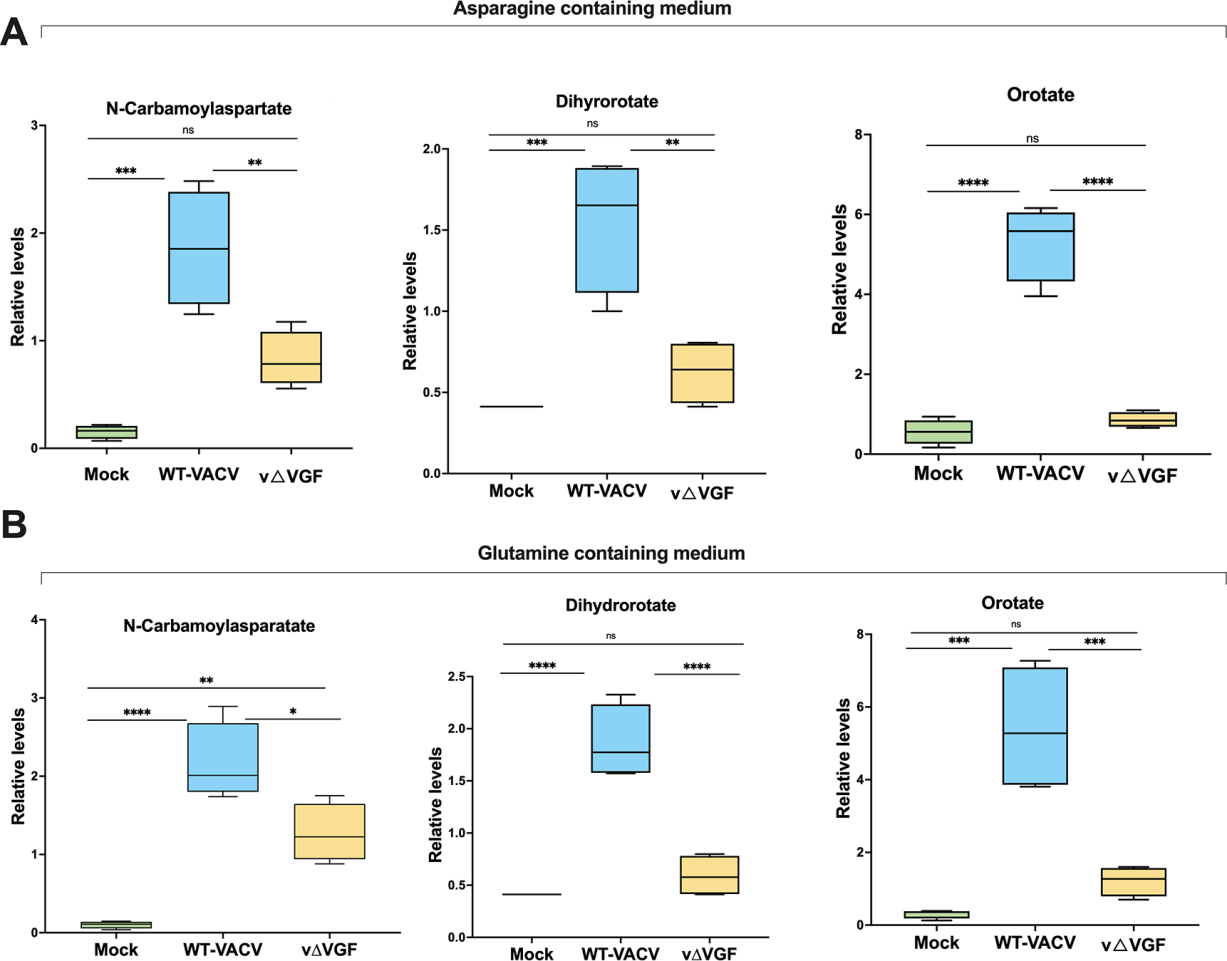
VACV infection induces elevated levels of intermediates of the *de novo* pyrimidine biosynthesis pathway in a VGF-dependent manner. (**A**) VGF is required to elevate the *de novo* pyrimidine pathway intermediates in asparagine-containing medium. The levels of metabolites measured by LC/MS at 8 hpi in HFFs cultured in media with glucose + asparagine. HFFs were infected with mock, WT-VACV, or v△VGF at an MOI of 3. (**B**) VGF is required to elevate the *de novo* pyrimidine pathway intermediates in glutamine-containing medium. The levels of metabolites measured by LC/MS at 8 hpi in HFFs cultured in a medium with glucose + glutamine with infections performed as in 3A. Statistical comparison between the three groups was performed using one-way ANOVA. ns, *P* > 0.05; *, P ≤ 0.05; **, P ≤ 0.01; ***, P ≤ 0.001; ****, P ≤ 0.001.

On analysis of the metabolic profiling in asparagine medium, we observed that infection with VACV markedly increased the levels of several key metabolites within the pyrimidine pathway when compared with mock-(uninfected) controls. Notably, there was an approximate 12-fold elevation in the levels of three pathway-specific metabolites: N-carbamoylaspartate, dihydroorotate, and orotate. Contrastingly, infection with vΔVGF, resulted in a significant decrease in the accumulation of these metabolites (Fig. 3A). Specifically, the levels of carbamoyl aspartate and dihydroorotate were reduced to approximately 2-fold, and orotate to around 7-fold lower than those observed under WT-VACV infection conditions. Interestingly, we noted similar trends in the levels of these intermediates upon infection in the glutamine-containing medium (Fig. 3B). Through these data, we show the necessary role of VGF in enhancing intermediates of the pyrimidine pathway upon VACV infection independent of glutamine or asparagine.

### VGF is necessary for the phosphorylation of CAD at serine1859 upon nutrient limitation

**Fig 4:**
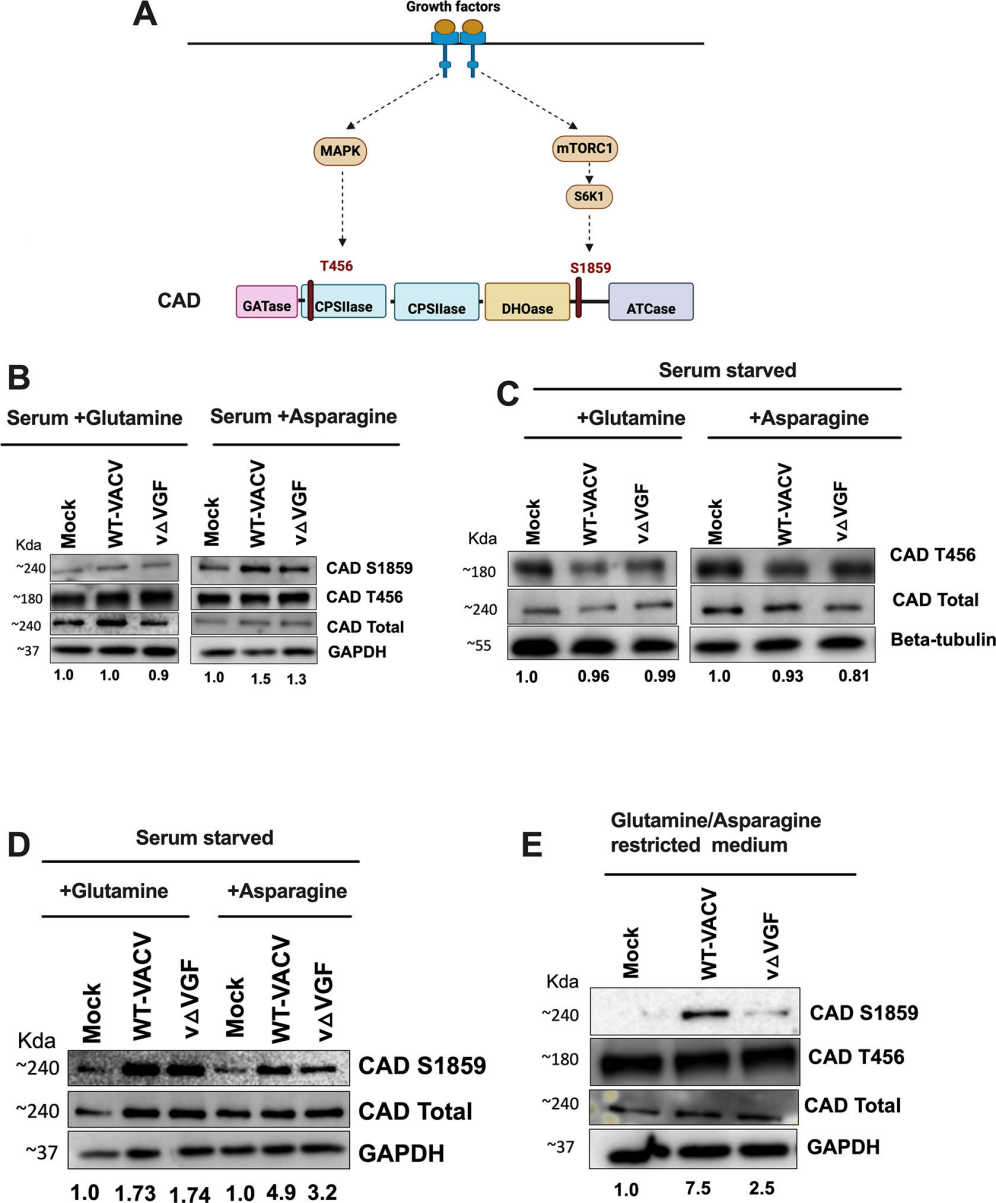
VGF activates CAD at S1859 in glutamine and asparagine-limited conditions. (**A**) Schematic representation of growth factor stimulation of CAD at two different phosphorylation sites. GATase: glutamine amidotransferase, CPSIIase: carbamoyl phosphate synthetase II, DHOase: dihydroorotase, ATCase: aspartate transcarbamoylase. (**B**) HFFs were mock-infected, infected with WT-VACV or v△VGF at an MOI of 5 with 2% dialyzed FBS, 2 mM Glutamine, or 2 mM Asparagine, and the cell lysates were collected for western blotting sample preparation at 8 hpi. (C and D) HFFs were starved with 0.1% dialyzed FBS and 2 mM glutamine or 2 mM asparagine for 48 h prior to infection. Infected cell lysates were collected at 8 hpi. For (**C**), samples from both conditions were loaded on the same SDS-PAGE gel. (**E**) HFFs were starved with 0.1% dialyzed FBS and 2 mM asparagine, followed by replacement with media containing 0.1% dialyzed FBS without glutamine or asparagine for approximately 16 h prior to infection. The infection media consisted of 0.1% dialyzed FBS without glutamine/asparagine. Infected cell lysates were collected at 8 hpi. Representative images from at least three biological replicates are shown. The numbers below the blots indicate the fold change of CAD S1859 (B, D, and E) and CAD T456 (**C**) based on the average intensities of Western blotting analysis from at least three biological replicates, with each replicate normalized to the loading control of its respective blot, and then further normalized to its mock treatment. Western blotting band quantification was determined by NIH Image J.

The CAD enzyme, a large multifunctional enzyme comprising approximately 2,225 amino acids, catalyzes the initial three rate-limiting reactions of the pyrimidine synthesis pathway. The enzymatic structure of CAD is differentiated into four distinct domains: glutamine amidotransferase (GATase), carbamoyl phosphate synthetase II (CPSIIase), dihydroorotase (DHOase), and aspartate transcarbamoylase (ATCase) (21, 22) (Fig. 4A). Furthermore, CAD has multiple regulatory sites and multiple amino acids of this protein can be phosphorylated by diverse signaling pathways, leading to distinct mechanisms of regulation for this large enzyme (46, 52). The two most extensively studied post-translational modifications of CAD are the phosphorylation at serine 1859 (located between the DHOase and ATCase domains) and threonine 456 (located in the CPSIIase domain). Both of these modifications are known to positively stimulate CAD activity (29, 53, 54) (Fig. 4A).

To determine how VGF regulates the *de novo* pyrimidine pathway, we compared the phosphorylation levels of these well-studied sites in uninfected cells, VACV-infected cells, and vΔVGF-infected cells. Phosphorylation of CAD at threonine 456 (T456) is facilitated by the MAPK signaling pathway upon activation of its upstream receptor EGFR, whereas phosphorylation at serine1859 (S1859) occurs through the activation of its upstream protein S6K1, which in turn is activated through the mTORC1 signaling pathway (21). Since it is well established that VGF stimulates the MAPK signaling pathway, we hypothesized that VGF is needed to modulate CAD phosphorylation at T456. We initially tested the role of VGF in media containing either glutamine or asparagine to ensure consistency with the experimental conditions employed in the metabolic profiling study. Upon examination, no significant differences were observed in the phosphorylation levels of CAD at T456 and S1859 between uninfected cells (mock), WT-VACV infection, and vΔVGF infection in a glutamine-containing medium. Although a minor increase in CAD phosphorylation levels was noted for S1849 for WT-VACV infection compared with the uninfected cells in the asparagine-containing medium, there was no significant difference between WT-VACV and vΔVGF infections under these specific conditions (Fig. 4B).

To eliminate the potential influence of confounding factors, present in fetal bovine serum (FBS) within the cell culture medium, on the function of VGF, HFFs were serum starved (~48 h) in either asparagine or glutamine-containing media. Upon serum starvation, consistent levels of T456 phosphorylation were observed across uninfected and infected (WT-VACV and vΔVGF) cells, in media supplemented with either glutamine or asparagine (Fig. 4C). Conversely, infection increased S1859 phosphorylation levels in the presence of either amino acid (Fig. 4D). These results suggest that asparagine or glutamine can independently facilitate CAD phosphorylation during VACV infection, potentially obfuscating the singular effects of discrete viral factors on this process. A moderate, albeit consistent, reduction in CAD S1859 phosphorylation was observed in the context of vΔVGF infection relative to WT-VACV infection, in serum-starved asparagine-containing medium (Fig. 4D). This observation also suggests that an asparagine-based medium may be suitable for establishing the role of VGF in regulating CAD.

Based on research conducted with cancer cells that demonstrated the activation of MAPK signaling as an adaptive mechanism upon asparagine restriction to sustain cancer proliferation, we treated HFF cells with an asparagine-containing medium and subsequently limited the glutamine and asparagine supply for ~16 h before infection. Upon examination of cells in this nutrient-restricted medium, it was observed that VACV infection significantly increased CAD S1859 phosphorylation by over 7.5-fold. In the context of vΔVGF infection, as opposed to WT-VACV infection, a notable reduction in CAD phosphorylation at S1859 was observed (Fig. 4E). This suggests that VGF positively regulates CAD at S1859 during glutamine/asparagine deprivation. Upon nutrient limitation, there was no noticeable change in CAD phosphorylation at T456. This indicates that VGF predominantly stimulates CAD via the S1859 site upon nutrient insufficiency (Fig. 4E). These findings highlight the function of VGF in the regulation of the key enzyme CAD in the *de novo* pyrimidine synthesis pathway upon nutrient stress, particularly in the context of glutamine and asparagine limitation. Conversely, no significant changes were observed in the protein expression levels of dihydroorotate dehydrogenase (DHODH), the subsequent essential enzyme of this pathway, across the conditions examined (data not presented).

### VACV activates CAD S1859 through mTORC1-S6K1 in a VGF-dependent manner

**Fig 5:**
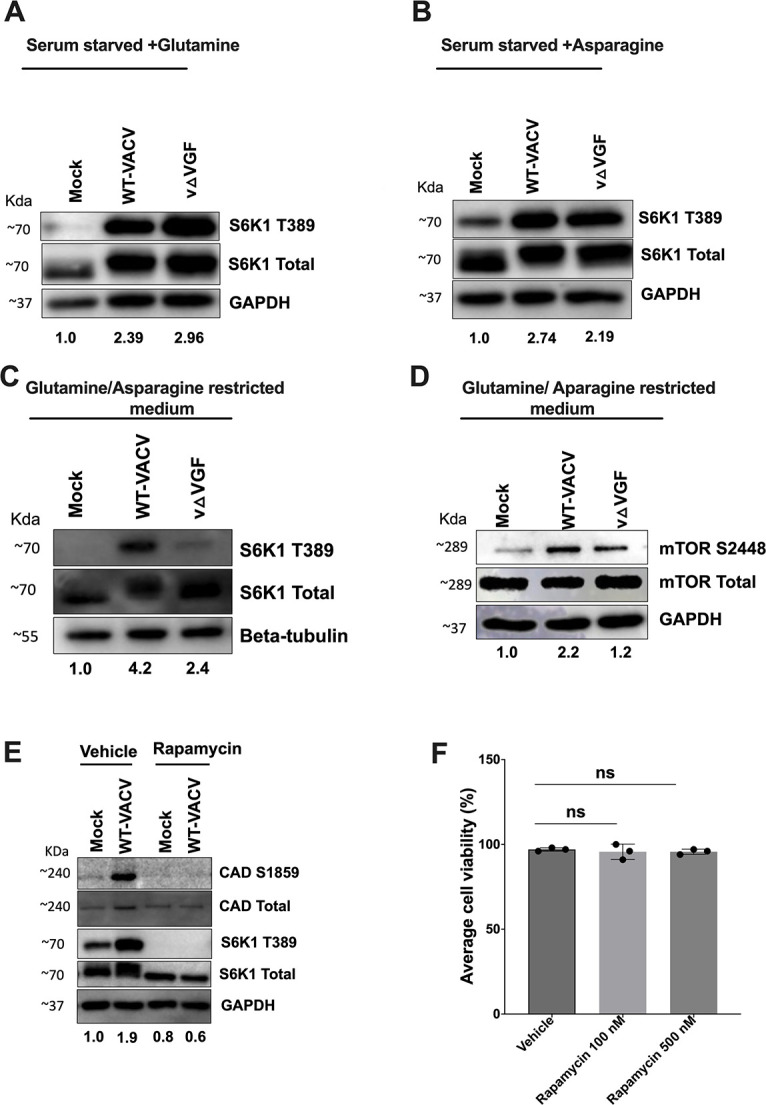
VGF is required to activate CAD through mTORC1-S6K1 upon VACV infection. (A and B) Role of VGF in activating mTORC1 downstream substrate S6K1 under different nutrient cues. HFFs were starved with 0.1% dialyzed FBS with either 2 mM glutamine (**A**) or 2 mM asparagine (**B**) for 48 h prior to infection. Cells were infected with indicated viruses at an MOI of 5 for 8 h. (C and D) HFFs were starved in media with 0.1% dialyzed FBS and 2 mM asparagine, followed by replacement with media containing 0.1% dialyzed FBS without glutamine or asparagine for approximately 16 h prior to infection. The infection media consisted of 0.1% dialyzed FBS without asparagine. Infection was carried out with indicated viruses using MOI 5 for 8 h. (**E**) HFFs were infected in conditions described in Fig. 4C and D. HFFs were either treated with vehicle (DMSO) or rapamycin (20 nM) one hpi and cell lysates were collected at 8 hpi. (**F**) HFFs were starved as described in Fig. 4C and D and treated with the indicated concentration of rapamycin for 24 h. following starvation. Cell viability was measured using trypan blue staining. Representative images from two (**D and E**) or three (A, B, C) biological replicates are shown. The numbers below the blot indicate the fold change of the indicated phosphorylated proteins based on the average intensities of Western blotting analyses from two (**D and E**) or three (**A, B, C**) biological replicates, with each replicate normalized to the loading control of its respective blot, and then further normalized to its mock treatment. Western blotting quantification was determined by NIH Image J.

We then aimed to elucidate the signaling pathway necessary for the phosphorylation of CAD at S1859 upon VACV infection through VGF. Previous investigations have established mTORC1 as an essential regulator of metabolic processes, capable of post-translationally modifying CAD to increase its enzymatic activity via phosphorylation at S1859 through the intermediary action of its downstream effector, S6K1 (50, 51). To examine the role of mTORC1 in the activation of CAD within the context of VACV infection, our initial approach involved assessing the necessity of VGF for mTORC1 signaling by examining the phosphorylation level of S6K1 (S6K1 T389) in glutamine or asparagine containing (serum-starved) media (52). Our observations of S6K1 phosphorylation in the presence of either glutamine or asparagine revealed a marked increase in S6K1 phosphorylation upon VACV infection. Notably, this enhancement exhibited no discernible difference between WT-VACV and vΔVGF infections (Fig. 5A and B). Conversely, upon glutamine and asparagine restriction (conditions same as Fig. 4E), a substantial reduction exceeding 2-fold in S6K1 phosphorylation levels was detected in cells infected with vΔVGF (Fig. 5C). Further validation of these findings was obtained through the observation of elevated mTORC1 S2448 phosphorylation in WT-VACV compared with vΔVGF infection (Fig. 5D). These outcomes collectively highlight that upon nutrient limitation, VGF is indispensable for the activation of S6K1, the downstream target of mTORC1 signaling.

Extending our investigation to determine the necessity of mTORC1-S6K1 activation for CAD phosphorylation at S1859, HFF cells were treated with the mTORC1 inhibitor rapamycin upon VACV-infection at a concentration that did not affect cell viability (53, 55). Rapamycin treatment resulted in a discernible reduction of CAD S1859 phosphorylation in WT-VACV conditions, as opposed to vehicle or untreated conditions (Fig. 5E and F). These findings indicate that upon nutrient restriction, particularly glutamine and asparagine, VACV is dependent on its viral factor VGF to modulate the mTORC1-CAD S1859 axis.

### Expression and purification of recombinant VGF peptide

**Fig 6:**
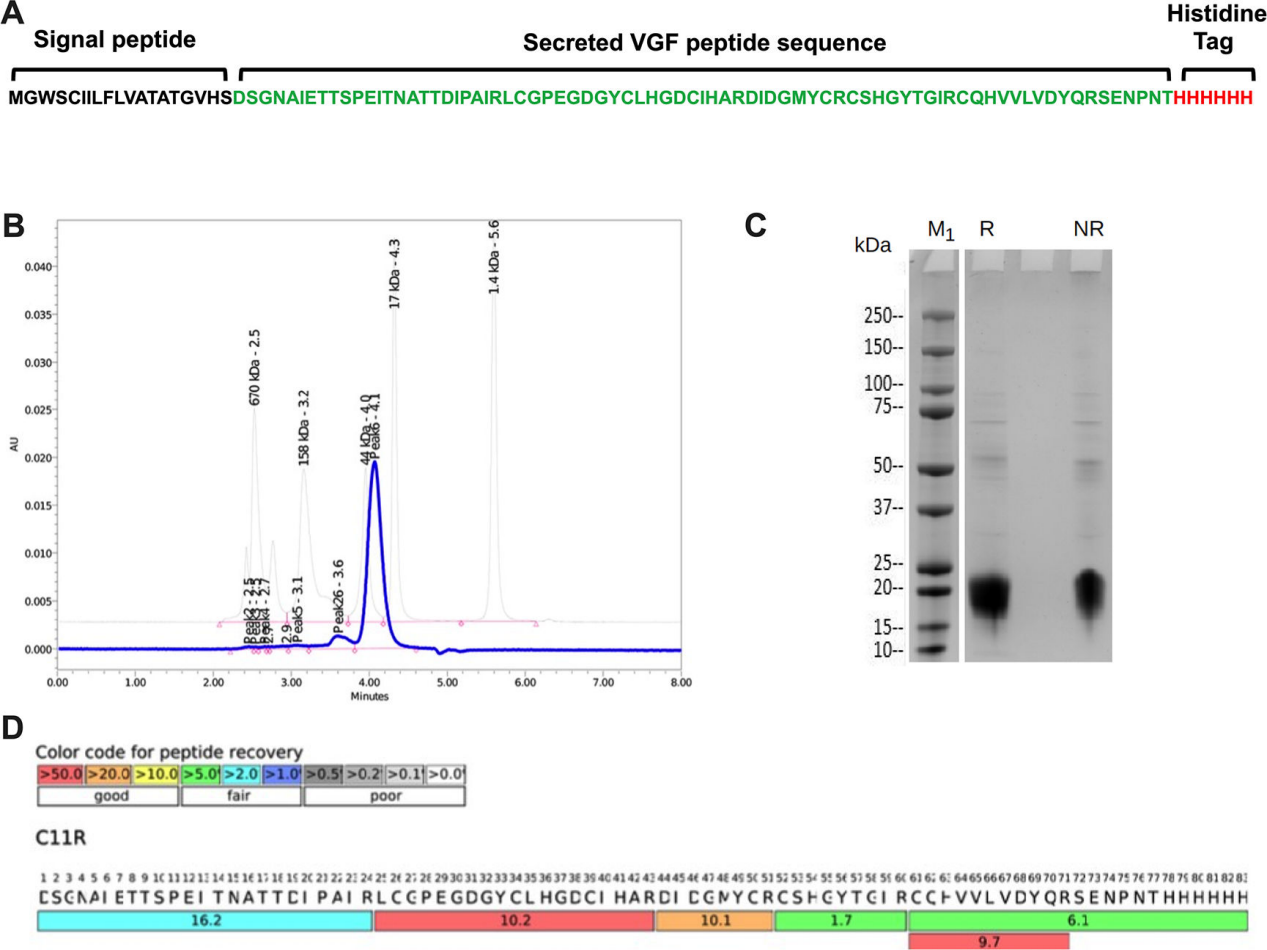
Expression, purification, and characterization of recombinant VGF peptide from a mammalian expression system. (A) Design of VGF sequence with the cleavable signal peptide at the N-terminal and a 6× Histidine tag at the C-terminal end. (B and C) Purified VGF peptide was examined through (B) SEC-HPLC and (C) SDS-PAGE by Coomassie blue staining. M1, protein marker; R, reducing condition; NR, non-reducing condition. (D) The sequence coverage was assessed with LC-MS after the purified VGF peptide was digested with trypsin.

To examine whether VGF, in the absence of VACV infection, could activate CAD and S6K1 signaling pathways, a recombinant peptide analogous to the processed, secreted variant of VGF was expressed in mammalian cells and purified. (Fig. 6A). The gene encoding VGF, denoted as C11R, was codon optimized, synthesized *de novo*, and subsequently cloned into the pcDNA3.4 plasmid vector. This construct was then expressed in mammalian CHO cells, to ensure appropriate post-translational modifications. To facilitate purification, a hexahistidine (6×His) tag was added to the peptide. Purification was achieved via size exclusion chromatography, with the peptide’s purity and molecular weight (~19 kDa) confirmed through SEC-HPLC (Fig. 6B) and SDS-PAGE followed by Coomassie Brilliant Blue staining (Fig. 6C), respectively. The peptide’s identity was further validated through sequence coverage after mass spectrometry (Fig. 6D).

### VGF peptide in the absence of VACV infection is insufficient to stimulate the mTORC1-CADS1859 axis

**Fig 7:**
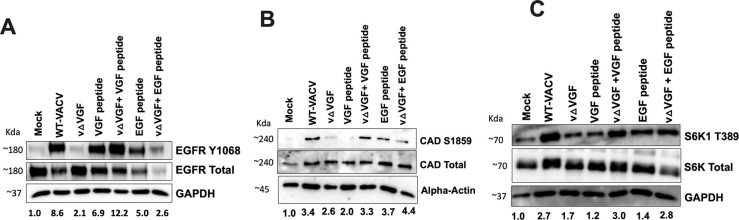
VGF peptide rescues vΔVGF reduction of CAD and S6K1 phosphorylation levels but not in the absence of infection. HFFs were starved with 0.1% dialyzed FBS in glutamine and asparagine-restricted medium as described in Fig. 4E. Cells were infected with indicated viruses at an MOI of 5. VGF or EGF peptide was added at the time of infection for respective samples at a concentration of 5 µg/mL. Cells were treated for a total of 8 h prior to sample preparation for western blotting analysis. EGFR (**A**), CAD S1859 (**B**), and S6K1 T389 (**C**) phosphorylation was revealed by western blotting analyses using indicated antibodies. Images presented are representative of three biological replicates. The numbers below the blot indicate the average fold change of the phosphorylated protein of interest based on three biological replicates, with each replicate normalized to the loading control of its respective blot, and then further normalized to its mock treatment. Western blotting band quantification was determined by NIH Image J.

We evaluated the efficacy of this recombinant VGF peptide in stimulating EGFR. Our observations indicated a negligible level of EGFR activation in both mock and vΔVGF conditions when compared with infections with WT-VACV, substantiating the essential role of VGF in EGFR activation (Fig. 7A). Treatment of HFFs with the VGF peptide alone resulted in EGFR stimulation. Notably, treatment of HFFs with the VGF peptide upon vΔVGF infection successfully rescued EGFR phosphorylation to levels comparable with those seen in WT-VACV infection. Conversely, although the EGF peptide independently activated EGFR, its treatment upon vΔVGF infection did not achieve full EGFR activation, reducing phosphorylation levels to those similar to the uninfected cells. Furthermore, the data imply that despite the homology between VGF and cellular EGF, the context of viral infection significantly enhances VGF’s ability to activate EGFR Y1068, suggesting that VGF and EGF exhibit divergent activities, likely attributable to the acquisition of unique functionalities of VGF by VACV. Interestingly, the treatment of HFF cells with VGF peptide in the absence of infection does not markedly increase the phosphorylation levels of CAD at S1859 and S6K1 T389 (Fig. 7B and C). The phosphorylation levels of both proteins when treated with the VGF peptide were found to be similar or only marginally elevated compared with the mock-treated cells. Conversely, the treatment of cells with EGF peptide alone, and combinations of vΔVGF with VGF or vΔVGF with EGF, rescued the mitigating effects induced by vΔVGF on the phosphorylation levels of CAD and S6K1. These outcomes indicate that in the context of vΔVGF infection, the VGF peptide can compensate for the absence of VGF for activation of CAD and mTORC1 signaling. Nonetheless, in the absence of such infection, the VGF peptide alone does not replicate these effects, indicating the requirement for additional viral factors/processes that synergize with VGF for the activation of CAD and S6K1 upon VACV infection. Intriguingly, an observed decrease in total EGFR levels upon WT-VACV infection or VGF/EGF treatment warrants further investigation.

## DISCUSSION

**Fig 8:**
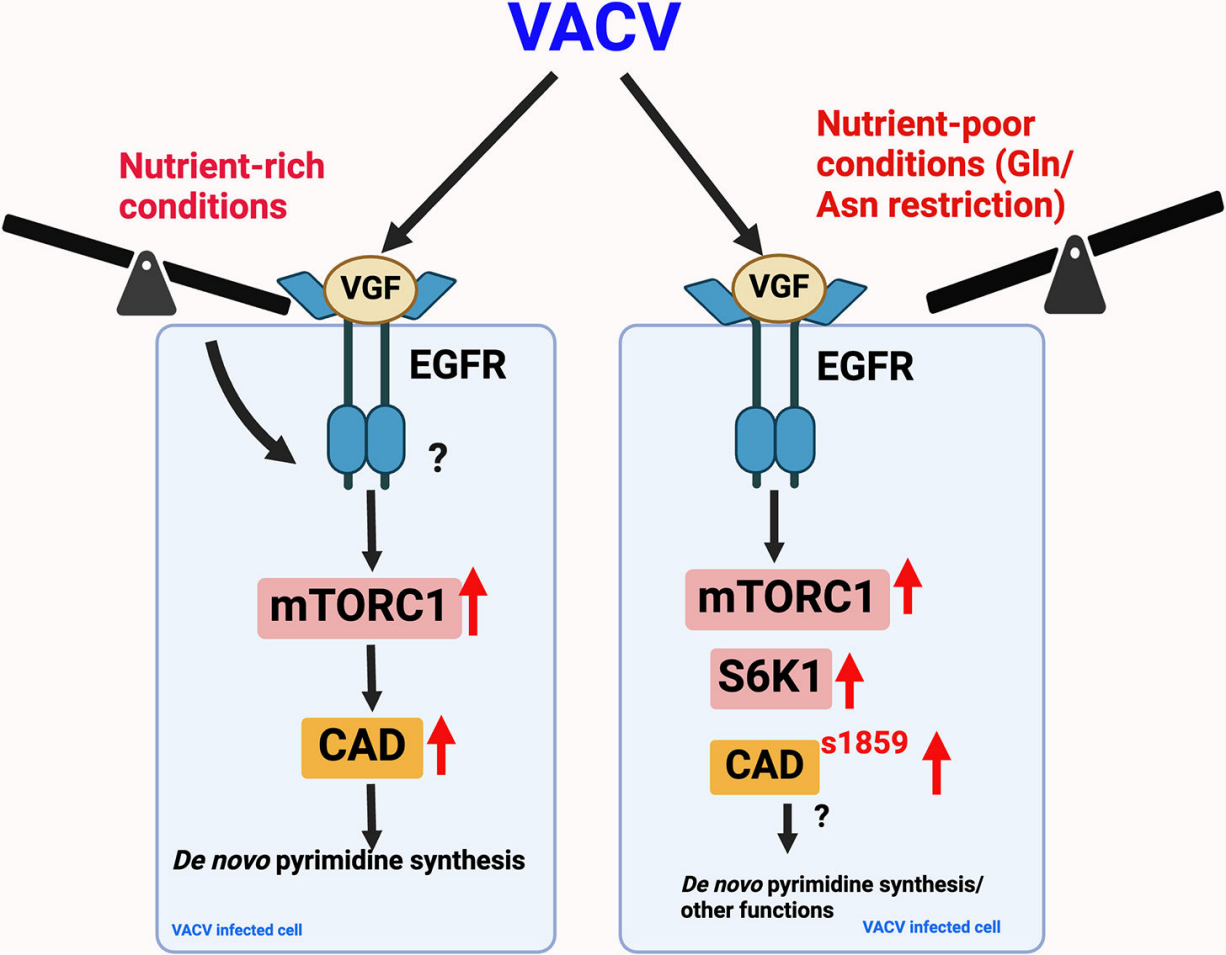
Proposed model of VACV’s regulation of the mTORC1-CAD axis under different nutrient cues. In nutrient-rich conditions, (glutamine or asparagine-containing media), CAD is activated independent of infection, thereby concealing the impact of VGF on the mTORC1-CAD axis. Conversely, upon nutrient deprivation (glutamine/asparagine limitation), VGF assumes a significant role in activating mTORC1 signaling to trigger CAD at serine1859, an important modulation necessary for the activation of the pyrimidine pathway.

Our study, in addition to highlighting the importance of the *de novo* pyrimidine pathway during VACV replication, also emphasizes the indispensability of the viral growth factor, VGF, in activating the mTORC1-S6K1 signaling axis and the subsequent phosphorylation of CAD at site S1859 upon nutrient insufficiency (Fig. 8). Our data reveal that in the presence of glutamine, or asparagine, upon infection, HFF cells maintain similar levels of CAD phosphorylation at S1859 and T456, independent of VGF. However, upon glutamine and asparagine limitation, VGF proves to be essential for the phosphorylation of CAD at site S1859 (Fig. 4). This highlights the nuanced regulatory mechanisms employed by VACV to ensure efficient replication, particularly upon nutrient insufficiency, highlighting the virus’s ability to manipulate host cellular pathways in response to different nutrient cues.

The regulation of CAD is complex, involving multiple post-translation modification sites. Specifically, T456, S1859, and S1873 are known to positively regulate CAD activity via the MAPK, mTORC1, and PKC pathways, respectively (22). Our data reveal consistent phosphorylation levels of CAD at T456 across different nutrient media (Fig. 4). This observation is suggestive that T456-mediated activation of CAD might be essential for sustaining basic cellular processes across varying nutritional environments, independent of infection. The VGF peptide, in isolation, is capable of activating the EGFR pathway; however, it cannot independently phosphorylate CAD and S6K1 in the absence of infection. Notably, the supplementation of the VGF peptide upon vΔVGF infection compensates for the reduced phosphorylation levels of CAD and S6K1 (Fig. 7). We report that infection by VACV requires additional viral factors beyond VGF for the activation of the mTORC1-CAD axis. Moreover, in contrast to VGF’s capacity to restore EGFR phosphorylation levels upon vΔVGF infection, the observed decrease in EGFR phosphorylation in the presence of EGF peptide upon vΔVGF infection is indicative that VGF, despite its homology to cellular EGF, is markedly more efficacious in mediating EGFR activation upon VACV infection. This observation could partially explain the ubiquitous encoding of VGF among almost all poxviruses. A comparison of amino acid between VGF and EGF shows that the two secreted peptides have nearly 50% difference in amino acid sequences, which provides an explanation for their evolutionary divergence, resulting in distinct functionalities. Additionally, VGF undergoes glycosylation as a post-translational modification, unlike EGF, which can further contribute to functional differences between the two proteins (7–9).

Given the critical role of EGFR in catalyzing key signaling cascades, these insights necessitate a detailed investigation into VGF-specific mechanisms for EGFR signaling activation and their consequent impacts upon infection. Furthermore, these findings imply a differential functionality of VGF as opposed to its cellular homolog. Elucidating the mechanisms through which VGF activates EGFR signaling could offer deeper insights into the mechanisms by which poxviruses alter key host signaling pathways.

CAD regulation includes acetylation, methylation, and ubiquitinylation beyond phosphorylation. Although limited, research implicates the roles of Rad9 and Rheb in CAD activation, with Rheb’s binding linked to increased nucleotide pools. Rheb activation via EGFR signaling has also been observed in tumorigenic contexts, suggesting VGF’s involvement in an alternative regulatory mechanism during nutrient sufficiency (56–59). Additionally, the *de novo* pyrimidine pathway supports nucleic acid synthesis and cellular processes, elevating UDP-GlcNAc, a key substrate for O-GlcNAcylation, which integrates nutritional and stress signals. VGF-dependent CAD phosphorylation at S1859 under nutrient limitation may further activate this pathway to increase UDP-GlcNAc levels (60).

In this study, we elucidate the mechanism by which CAD phosphorylation S1859 is activated through the phosphorylation of S6K1 at T389, a known substrate of the mTORC1 complex (Fig. 5), especially upon nutrient restriction during infection. mTORC1, established as a central regulator of cellular metabolism, orchestrates the integration of signals from various nutrients and growth factors to modulate both anabolic and catabolic processes (61). It is noteworthy that complete mTORC1 activation is unattainable through growth factor signaling alone; it necessitates concurrent amino acid stimulation. Independent activation of mTORC1 signaling by glutamine and asparagine has been reported, suggesting that despite serum starvation, these amino acids are utilized by VACV and are sufficient to activate mTORC1 during infection, independent of VGF (62).

However, VACV’s sustained activation of mTORC1 upon glutamine/asparagine deprivation suggests an adaptive mechanism, wherein VGF plays a crucial role in facilitating viral replication under conditions of nutrient insufficiency. Amino acid limitation inactivates mTORC1 activity, subsequently inhibiting cellular growth and proliferation (63). Prior research has reported the sustenance of mTORC1 activity by herpes simplex virus and human cytomegalovirus upon nutrient insufficiency, providing insights into viral regulatory mechanisms upon physiological stress (64, 65). The ability of poxviruses to activate mTORC1 upon nutrient stress was previously unknown. Our findings now reveal a mechanism through which VACV through its viral protein, VGF, adapts to nutrient stress.

The dual requirement of amino acids and growth factors for maximal mTORC1 activation suggests that VACV, along with VGF, may engage additional survival mechanisms for nutrient acquisition under glutamine/asparagine limitation. In cancer cells, MAPK signaling activates ATF4 during asparagine restriction, regulating genes involved in amino acid metabolism and stress response (35). The mTORC1-ATF4 axis also governs genes related to amino acid uptake and synthesis (66). This implies that VACV, via VGF, could exploit this axis to regulate nutrient uptake during nutrient limitation, warranting further investigation.

It has been reported that F17, synthesized during the late stages of VACV infection, disrupts mTORC1 activity (67). Given that F17 is a virion core protein likely transferred to the host cell upon entry, the presence of F17 alone does not substantially activate mTORC1 within the initial 8 h post-infection upon asparagine restriction, as evidenced by the absence of significant S6K1 activation in cells infected with vΔVGF. This suggests that VACV may leverage different viral proteins to activate specific signaling pathways in various nutritional and physiological contexts or that VGF and F17 might act synergistically to modulate mTORC1 signaling.

VACV has been utilized as an oncolytic agent for treating cancers, with emerging evidence supporting the benefits of prolonged starvation in increasing oncolytic virotherapy efficacy for cancer treatment (39, 68). Our findings underscore the crucial role of VGF in activating mTORC1 during nutrient deprivation, revealing how VACV shapes metabolism in specific physiological contexts. These insights carry significant potential for improving the effectiveness of VACV as an oncolytic agent in cancer therapy.

Additionally, the variable nutritional environments encountered by tumor growth, characterized by distinct amino acid vulnerabilities in different tumors further necessitate a deeper understanding of how VACV adapts its signaling mechanisms to diverse nutritional cues, potentially advancing its therapeutic application as an oncolytic agent (66, 69).

The requirement of nucleotides for efficient viral replication renders nucleotide synthesis inhibition a promising antiviral therapeutic strategy (70). Nevertheless, the context-specific mechanisms employed by different viruses to sustain replication highlight the complexity of targeting such pathways. The current challenges posed by existing CAD inhibitors due to off-target effects underscore the need for more potent and specific inhibitors of this enzyme (71). Our study uncovers a novel mechanism of mTORC1 regulation via VGF upon infection. By identifying the specific regulatory sites of CAD essential for its activation during VACV infection, we aim to fill critical gaps in understanding this key rate-limiting enzyme, with implications for poxvirus-specific therapies. Our findings suggest the potential for more precise therapeutic strategies aimed not only at nucleotide inhibition but also at inhibiting upstream signaling pathways.

Overall, we demonstrate the indispensable function of the viral early protein, VGF, in mediating CAD activation at S1859, specifically upon nutrient restriction (Fig. 8). Our findings highlight that despite glutamine and asparagine limitation during VACV infection, VGF signaling activates mTORC1, leading to the activation of its downstream effector CAD. This process is crucial for the induction of the *de novo* pyrimidine synthesis pathway. These insights contribute to our overall understanding of the molecular interplay between VACV infection and host cell metabolic regulation, potentially opening avenues for targeted therapeutic interventions.

## Data Availability

The data include Western blotting analyses, virus titration, PCR, luciferase luminescence activities, and relative metabolite levels. The authors confirm that the data supporting the findings of this study are included within the article, and the raw data can be provided upon request. This paper does not include any large data sets.

## References

[1] 2024. Future state of smallpox medical countermeasures. National Academies Press, Washington, D.C. 39078926

[2] Durski KN, McCollum AM, Nakazawa Y, Petersen BW, Reynolds MG, Briand S, Djingarey MH, Olson V, Damon IK, Khalakdina A. 2018. Emergence of monkeypox — West and Central Africa, 1970–2017. MMWR 67:306–310. doi:10.15585/mmwr.mm6710a5 29543790 PMC5857192

[3] Yang Z. 2022. Monkeypox: a potential global threat? J Med Virol 94:4034–4036. doi:10.1002/jmv.27884 35614026 PMC9283296

[4] Yang Z, Gray M, Winter L. 2021. Why do poxviruses still matter? Cell Biosci 11:96. doi:10.1186/s13578-021-00610-8 34022954 PMC8140567

[5] Verardi PH, Titong A, Hagen CJ. 2012. A vaccinia virus renaissance. Hum Vaccin Immunother 8:961–970. doi:10.4161/hv.21080 22777090 PMC3495727

[6] Hendrickson RC, Wang C, Hatcher EL, Lefkowitz EJ. 2010. Orthopoxvirus genome evolution: the role of gene loss. Viruses 2:1933–1967. doi:10.3390/v2091933 21994715 PMC3185746

[7] Chang W, Lim JG, Hellström I, Gentry LE. 1988. Characterization of vaccinia virus growth factor biosynthetic pathway with an antipeptide antiserum. J Virol 62:1080–1083. doi:10.1128/JVI.62.3.1080-1083.1988 3339713 PMC253672

[8] Blomquist MC, Hunt LT, Barker WC. 1984. Vaccinia virus 19-kilodalton protein: relationship to several mammalian proteins, including two growth factors. Proc Natl Acad Sci U S A 81:7363–7367. doi:10.1073/pnas.81.23.7363 6334307 PMC392146

[9] Twardzik DR, Brown JP, Ranchalis JE, Todaro GJ, Moss B. 1985. Vaccinia virus-infected cells release a novel polypeptide functionally related to transforming and epidermal growth factors. Proc Natl Acad Sci U S A 82:5300–5304. doi:10.1073/pnas.82.16.5300 3875097 PMC390555

[10] Yang Z, Bruno DP, Martens CA, Porcella SF, Moss B. 2010. Simultaneous high-resolution analysis of vaccinia virus and host cell transcriptomes by deep RNA sequencing. Proc Natl Acad Sci U S A 107:11513–11518. doi:10.1073/pnas.1006594107 20534518 PMC2895082

[11] Beerli C, Yakimovich A, Kilcher S, Reynoso GV, Fläschner G, Müller DJ, Hickman HD, Mercer J. 2019. Vaccinia virus hijacks EGFR signalling to enhance virus spread through rapid and directed infected cell motility. Nat Microbiol 4:216–225. doi:10.1038/s41564-018-0288-2 30420785 PMC6354922

[12] Goodwin CM, Xu S, Munger J. 2015. Stealing the keys to the kitchen: viral manipulation of the host cell metabolic network. Trends Microbiol 23:789–798. doi:10.1016/j.tim.2015.08.007 26439298 PMC4679435

[13] Thaker SK, Ch’ng J, Christofk HR. 2019. Viral hijacking of cellular metabolism. BMC Biol 17:59. doi:10.1186/s12915-019-0678-9 31319842 PMC6637495

[14] Sanchez EL, Lagunoff M. 2015. Viral activation of cellular metabolism. Virology (Auckl) 479–480:609–618. doi:10.1016/j.virol.2015.02.038 PMC4424078 25812764

[15] Fontaine KA, Camarda R, Lagunoff M. 2014. Vaccinia virus requires glutamine but not glucose for efficient replication. J Virol 88:4366–4374. doi:10.1128/JVI.03134-13 24501408 PMC3993723

[16] Greseth MD, Traktman P. 2014. De novo fatty acid biosynthesis contributes significantly to establishment of a bioenergetically favorable environment for vaccinia virus infection. PLoS Pathog 10:e1004021. doi:10.1371/journal.ppat.1004021 24651651 PMC3961357

[17] Pant A, Brahim Belhaouari D, Dsouza L, Yang Z. 2024. Stimulation of neutral lipid synthesis via viral growth factor signaling and ATP citrate lyase during vaccinia virus infection. J Virol 98:e0110324. doi:10.1128/jvi.01103-24 39475274 PMC11578090

[18] Pant A, Dsouza L, Cao S, Peng C, Yang Z. 2021. Viral growth factor- and STAT3 signaling-dependent elevation of the TCA cycle intermediate levels during vaccinia virus infection. PLoS Pathog 17:e1009303. doi:10.1371/journal.ppat.1009303 33529218 PMC7880457

[19] Sprenger H-G, MacVicar T, Bahat A, Fiedler KU, Hermans S, Ehrentraut D, Ried K, Milenkovic D, Bonekamp N, Larsson N-G, Nolte H, Giavalisco P, Langer T. 2021. Cellular pyrimidine imbalance triggers mitochondrial DNA-dependent innate immunity. Nat Metab 3:636–650. doi:10.1038/s42255-021-00385-9 33903774 PMC8144018

[20] Sahu U, Villa E, Reczek CR, Zhao Z, O’Hara BP, Torno MD, Mishra R, Shannon WD, Asara JM, Gao P, Shilatifard A, Chandel NS, Ben-Sahra I. 2024. Pyrimidines maintain mitochondrial pyruvate oxidation to support de novo lipogenesis. Science 383:1484–1492. doi:10.1126/science.adh2771 38547260 PMC11325697

[21] Li G, Li D, Wang T, He S. 2021. Pyrimidine biosynthetic enzyme CAD: its function, regulation, and diagnostic potential. Int J Mol Sci 22:10253. doi:10.3390/ijms221910253 34638594 PMC8508918

[22] Del Caño-Ochoa F, Moreno-Morcillo M, Ramón-Maiques S. 2019. CAD, a multienzymatic protein at the head of *de novo* pyrimidine biosynthesis, p 505–538. In Harris JR, Marles-Wright J (ed), Macromolecular protein complexes II: structure and function. Vol. 93. Springer International Publishing, Cham. 10.1007/978-3-030-28151-9_17 31939163

[23] Walter M, Herr P. 2022. Re-discovery of pyrimidine salvage as target in cancer therapy. Cells 11:739. doi:10.3390/cells11040739 35203388 PMC8870348

[24] Lane AN, Fan TW-M. 2015. Regulation of mammalian nucleotide metabolism and biosynthesis. Nucleic Acids Res 43:2466–2485. doi:10.1093/nar/gkv047 25628363 PMC4344498

[25] Valle-Casuso J-C, Gaudaire D, Martin-Faivre L, Madeline A, Dallemagne P, Pronost S, Munier-Lehmann H, Zientara S, Vidalain P-O, Hans A. 2020. Replication of equine arteritis virus is efficiently suppressed by purine and pyrimidine biosynthesis inhibitors. Sci Rep 10:10100. doi:10.1038/s41598-020-66944-4 32572069 PMC7308276

[26] Tang N, Chen P, Zhao C, Liu P, Tan L, Song C, Qiu X, Liao Y, Liu X, Luo T, Sun Y, Ding C. 2023. Newcastle disease virus manipulates mitochondrial MTHFD2-mediated nucleotide metabolism for virus replication. J Virol 97:e0001623. doi:10.1128/jvi.00016-23 36794935 PMC10062132

[27] DeVito SR, Ortiz-Riaño E, Martínez-Sobrido L, Munger J. 2014. Cytomegalovirus-mediated activation of pyrimidine biosynthesis drives UDP-sugar synthesis to support viral protein glycosylation. Proc Natl Acad Sci U S A 111:18019–18024. doi:10.1073/pnas.1415864111 25472841 PMC4273352

[28] Katsafanas GC, Grem JL, Blough HA, Moss B. 1997. Inhibition of vaccinia virus replication by N-(phosphonoacetyl)-l-aspartate: differential effects on viral gene expression result from a reduced pyrimidine nucleotide pool. Virology (Auckl) 236:177–187. doi:10.1006/viro.1997.8735 9299630

[29] Sigoillot FD, Kotsis DH, Serre V, Sigoillot SM, Evans DR, Guy HI. 2005. Nuclear localization and mitogen-activated protein kinase phosphorylation of the multifunctional protein CAD. J Biol Chem 280:25611–25620. doi:10.1074/jbc.M504581200 15890648

[30] Evans DR, Guy HI. 2004. Mammalian pyrimidine biosynthesis: fresh insights into an ancient pathway. J Biol Chem 279:33035–33038. doi:10.1074/jbc.R400007200 15096496

[31] Sigoillot FD, Kotsis DH, Masko EM, Bame M, Evans DR, Evans HIG. 2007. Protein kinase C modulates the up-regulation of the pyrimidine biosynthetic complex, CAD, by MAP kinase. Front Biosci 12:3892–3898. doi:10.2741/2358 17485345

[32] Pant A, Cao S, Yang Z. 2019. Asparagine is a critical limiting metabolite for vaccinia virus protein synthesis during glutamine deprivation. J Virol 93:e01834-18. doi:10.1128/JVI.01834-18 30996100 PMC6580962

[33] Yoo HC, Yu YC, Sung Y, Han JM. 2020. Glutamine reliance in cell metabolism. Exp Mol Med 52:1496–1516. doi:10.1038/s12276-020-00504-8 32943735 PMC8080614

[34] Pathria G, Lee JS, Hasnis E, Tandoc K, Scott DA, Verma S, Feng Y, Larue L, Sahu AD, Topisirovic I, Ruppin E, Ronai ZA. 2019. Translational reprogramming marks adaptation to asparagine restriction in cancer. Nat Cell Biol 21:1590–1603. doi:10.1038/s41556-019-0415-1 31740775 PMC7307327

[35] Zhang J, Fan J, Venneti S, Cross JR, Takagi T, Bhinder B, Djaballah H, Kanai M, Cheng EH, Judkins AR, Pawel B, Baggs J, Cherry S, Rabinowitz JD, Thompson CB. 2014. Asparagine plays a critical role in regulating cellular adaptation to glutamine depletion. Mol Cell 56:205–218. doi:10.1016/j.molcel.2014.08.018 25242145 PMC4224619

[36] Pavlova NN, Hui S, Ghergurovich JM, Fan J, Intlekofer AM, White RM, Rabinowitz JD, Thompson CB, Zhang J. 2018. As extracellular glutamine levels decline, asparagine becomes an essential amino acid. Cell Metab 27:428–438. doi:10.1016/j.cmet.2017.12.006 29337136 PMC5803449

[37] Szwed A, Kim E, Jacinto E. 2021. Regulation and metabolic functions of mTORC1 and mTORC2. Physiol Rev 101:1371–1426. doi:10.1152/physrev.00026.2020 33599151 PMC8424549

[38] Saxton RA, Sabatini DM. 2017. mTOR signaling in growth, metabolism, and disease. Cell 168:960–976. doi:10.1016/j.cell.2017.02.004 28283069 PMC5394987

[39] Li M, Zhang M, Ye Q, Liu Y, Qian W. 2023. Preclinical and clinical trials of oncolytic vaccinia virus in cancer immunotherapy: a comprehensive review. Cancer Biol Med 20:646–661. doi:10.20892/j.issn.2095-3941.2023.0202 37615308 PMC10546091

[40] Yaghchi CA, Zhang Z, Alusi G, Lemoine NR, Wang Y. 2015. Vaccinia virus, a promising new therapeutic agent for pancreatic cancer. Immunother (Los Angel) 7:1249–1258. doi:10.2217/imt.15.90 PMC4976866 26595180

[41] Cotter CA, Earl PL, Wyatt LS, Moss B. 2017. Preparation of cell cultures and vaccinia virus stocks. Curr Protoc Protein Sci 89:5. doi:10.1002/cpps.34 28762495

[42] Baer A, Kehn-Hall K. 2014. Viral concentration determination through plaque assays: using traditional and novel overlay systems. J Vis Exp 52065:e52065. doi:10.3791/52065 PMC4255882 25407402

[43] Strober W. 2015. Trypan blue exclusion test of cell viability. Curr Protoc Immunol 111:A3. doi:10.1002/0471142735.ima03bs111 PMC6716531 26529666

[44] Gibson SJ, Bond NJ, Milne S, Lewis A, Sheriff A, Pettman G, Pradhan R, Higazi DR, Hatton D. 2017. N-terminal or signal peptide sequence engineering prevents truncation of human monoclonal antibody light chains. Biotechnol Bioeng 114:1970–1977. doi:10.1002/bit.26301 28369727

[45] O’Neill P, Mistry RK, Brown AJ, James DC. 2023. Protein-specific signal peptides for mammalian vector engineering. ACS Synth Biol 12:2339–2352. doi:10.1021/acssynbio.3c00157 37487508 PMC10443038

[46] Huang M, Graves LM. 2003. De novo synthesis of pyrimidine nucleotides; emerging interfaces with signal transduction pathways. Cell Mol Life Sci 60:321–336. doi:10.1007/s000180300027 12678497 PMC11146060

[47] Ringer DP, Howell BA, Etheredge JL. 1991. Alteration in de novo pyrimidine biosynthesis during uridine reversal of pyrazofurin-inhibited DNA synthesis. J Biochem Toxicol 6:19–27. doi:10.1002/jbt.2570060104 1880786

[48] Greene S, Watanabe K, Braatz-Trulson J, Lou L. 1995. Inhibition of dihydroorotate dehydrogenase by the immunosuppressive agent leflunomide. Biochem Pharmacol 50:861–867. doi:10.1016/0006-2952(95)00255-x 7575649

[49] Teschner S, Burst V. 2010. Leflunomide: a drug with a potential beyond rheumatology. Immunotherapy (Los Angel) 2:637–650. doi:10.2217/imt.10.52 20874647

[50] Cadman EC, Dix DE, Handschumacher RE. 1978. Clinical, biological, and biochemical effect of pyrazofurin. Cancer Res 38:682–688. 272228

[51] Greseth MD, Traktman P. 2022. The life cycle of the vaccinia virus genome. Annu Rev Virol 9:239–259. doi:10.1146/annurev-virology-091919-104752 35584888

[52] Villa E, Ali ES, Sahu U, Ben-Sahra I. 2019. Cancer cells tune the signaling pathways to empower de novo synthesis of nucleotides. Cancers (Basel) 11:688. doi:10.3390/cancers11050688 31108873 PMC6562601

[53] Robitaille AM, Christen S, Shimobayashi M, Cornu M, Fava LL, Moes S, Prescianotto-Baschong C, Sauer U, Jenoe P, Hall MN. 2013. Quantitative phosphoproteomics reveal mTORC1 activates de novo pyrimidine synthesis. Science 339:1320–1323. doi:10.1126/science.1228771 23429704

[54] Ben-Sahra I, Howell JJ, Asara JM, Manning BD. 2013. Stimulation of de novo pyrimidine synthesis by growth signaling through mTOR and S6K1. Science 339:1323–1328. doi:10.1126/science.1228792 23429703 PMC3753690

[55] Lamming DW. 2016. Inhibition of the mechanistic target of rapamycin (mTOR)-rapamycin and beyond. Cold Spring Harb Perspect Med 6:a025924. doi:10.1101/cshperspect.a025924 27048303 PMC4852795

[56] Uribe ML, Marrocco I, Yarden Y. 2021. EGFR in cancer: signaling mechanisms, drugs, and acquired resistance. Cancers (Basel) 13:2748. doi:10.3390/cancers13112748 34206026 PMC8197917

[57] Lindsey-Boltz LA, Wauson EM, Graves LM, Sancar A. 2004. The human Rad9 checkpoint protein stimulates the carbamoyl phosphate synthetase activity of the multifunctional protein CAD. Nucleic Acids Res 32:4524–4530. doi:10.1093/nar/gkh789 15326225 PMC516061

[58] Sato T, Akasu H, Shimono W, Matsu C, Fujiwara Y, Shibagaki Y, Heard JJ, Tamanoi F, Hattori S. 2015. Rheb protein binds CAD (carbamoyl-phosphate synthetase 2, aspartate transcarbamoylase, and dihydroorotase) protein in a GTP- and effector domain-dependent manner and influences its cellular localization and carbamoyl-phosphate synthetase (CPSase) activity. J Biol Chem 290:1096–1105. doi:10.1074/jbc.M114.592402 25422319 PMC4294477

[59] Lee MJ, Yaffe MB. 2016. Protein regulation in signal transduction. Cold Spring Harb Perspect Biol 8:a005918. doi:10.1101/cshperspect.a005918 27252361 PMC4888820

[60] Liu Y, Yao R-Z, Lian S, Liu P, Hu Y-J, Shi H-Z, Lv H-M, Yang Y-Y, Xu B, Li S-Z. 2021. O-GlcNAcylation: the “stress and nutrition receptor” in cell stress response. Cell Stress Chaperones 26:297–309. doi:10.1007/s12192-020-01177-y 33159661 PMC7925768

[61] Goul C, Peruzzo R, Zoncu R. 2023. The molecular basis of nutrient sensing and signalling by mTORC1 in metabolism regulation and disease. Nat Rev Mol Cell Biol 24:857–875. doi:10.1038/s41580-023-00641-8 37612414

[62] Meng D, Yang Q, Wang H, Melick CH, Navlani R, Frank AR, Jewell JL. 2020. Glutamine and asparagine activate mTORC1 independently of Rag GTPases. J Biol Chem 295:2890–2899. doi:10.1074/jbc.AC119.011578 32019866 PMC7062167

[63] Liu GY, Sabatini DM. 2020. mTOR at the nexus of nutrition, growth, ageing and disease. Nat Rev Mol Cell Biol 21:183–203. doi:10.1038/s41580-019-0199-y 31937935 PMC7102936

[64] Vink EI, Lee S, Smiley JR, Mohr I. 2018. Remodeling mTORC1 responsiveness to amino acids by the herpes simplex virus UL46 and Us3 gene products supports replication during nutrient insufficiency. J Virol 92:e01377-18. doi:10.1128/JVI.01377-18 30282708 PMC6258953

[65] Clippinger AJ, Maguire TG, Alwine JC. 2011. Human cytomegalovirus infection maintains mTOR activity and its perinuclear localization during amino acid deprivation. J Virol 85:9369–9376. doi:10.1128/JVI.05102-11 21734039 PMC3165763

[66] Pathria G, Ronai ZA. 2021. Harnessing the co-vulnerabilities of amino acid-restricted cancers. Cell Metab 33:9–20. doi:10.1016/j.cmet.2020.12.009 33406406 PMC7837405

[67] Meade N, King M, Munger J, Walsh D. 2019. mTOR dysregulation by vaccinia virus F17 controls multiple processes with varying roles in infection. J Virol 93:e00784-19. doi:10.1128/JVI.00784-19 31118254 PMC6639273

[68] Scheubeck G, Berchtold S, Smirnow I, Schenk A, Beil J, Lauer UM. 2019. Starvation-induced differential virotherapy using an oncolytic measles vaccine virus. Viruses 11:614. doi:10.3390/v11070614 31284426 PMC6669668

[69] Lyssiotis CA, Kimmelman AC. 2017. Metabolic interactions in the tumor microenvironment. Trends Cell Biol 27:863–875. doi:10.1016/j.tcb.2017.06.003 28734735 PMC5814137

[70] Ariav Y, Ch’ng JH, Christofk HR, Ron-Harel N, Erez A. 2021. Targeting nucleotide metabolism as the nexus of viral infections, cancer, and the immune response. Sci Adv 7:eabg6165. doi:10.1126/sciadv.abg6165 34138729 PMC8133749

[71] Wang W, Cui J, Ma H, Lu W, Huang J. 2021. Targeting pyrimidine metabolism in the era of precision cancer medicine. Front Oncol 11:684961. doi:10.3389/fonc.2021.684961 34123854 PMC8194085

